# Geographic Variation and Associated Covariates of Diabetes Prevalence in India

**DOI:** 10.1001/jamanetworkopen.2020.3865

**Published:** 2020-05-01

**Authors:** Andrés M. Hernandez, Peng Jia, Hae-Young Kim, Diego F. Cuadros

**Affiliations:** 1 Department of Geography and Geographic Information Science, University of Cincinnati, Cincinnati, Ohio; 2 Health Geography and Disease Modeling Laboratory, University of Cincinnati, Cincinnati, Ohio; 3 Department of Land Surveying and Geo-Informatics, The Hong Kong Polytechnic University, Hong Kong, China; 4 State Key Laboratory of Urban and Regional Ecology, Research Center for Eco-Environmental Sciences, Chinese Academy of Sciences, Beijing, China; 5 Africa Health Research Institute, Kwazulu-Natal, South Africa; 6 University of KwaZulu-Natal School of Nursing and Public Health, Kwazulu-Natal, South Africa

## Abstract

**Importance:**

Diabetes is a severe metabolic disorder affecting human health worldwide, with increasing prevalence in low- and middle-income countries. Gaps in knowledge regarding factors that lead to diabetes and its association with tuberculosis (TB) endemicity at the national scale still exist, mainly because of the lack of large-scale dual testing and appropriate evaluation methods.

**Objectives:**

To identify locations in India where diabetes prevalence is concentrated, examine the association of diabetes with sociodemographic and behavioral covariates, and uncover where high regional TB endemicity overlaps with diabetes.

**Design, Setting, and Participants:**

This cross-sectional study included 803 164 men aged 15 to 54 years and women aged 15 to 49 years who participated in the Demographic Health Survey (2015-2016), carried out by the India Ministry of Health and Family Welfare using a 2-stage clustered sampling, which included a diabetes estimation component. The survey was conducted from January 2015 to December 2016, and data analysis was conducted from July 2018 to January 2019.

**Exposures:**

Self-reported diabetes status.

**Main Outcomes and Measures:**

Self-reported diabetes status was used to estimate the association of covariates, including educational level, sex, age, religion, marital status, alcohol use, tobacco use, obesity status, and household socioeconomic level, with diabetes prevalence. Additionally, regional tuberculosis endemicity level, estimated using the India TB report for 2014 from the Revised National TB Program, was included to evaluate the national extent of the spatial overlap of diabetes and TB.

**Results:**

Among 803 164 sampled individuals (691 982 [86.2%] women; mean [SD] age, 30.09 [9.97] years), substantial geographic variation in diabetes prevalence in India was found, with a concentrated burden at the southern coastline (cluster 1, Andhra Pradesh and Telangana: prevalence, 3.01% [1864 of 61 948 individuals]; cluster 2, Tamil Nadup and Kerala: prevalence, 4.32% [3429 of 79 435 individuals]; cluster 3, east Orissa: prevalence, 2.81% [330 of 11 758 individuals]; cluster 4, Goa: prevalence, 4.43% [83 of 1883 individuals]). Having obesity and overweight (odds ratio [OR], 2.44; 95% CI, 2.18-2.73; *P* < .001; OR, 1.66; 95% CI, 1.52-1.82; *P* < .001, respectively), smoking tobacco (OR, 3.04; 95% CI, 1.66-5.56; *P* < .001), and consuming alcohol (OR, 2.01; 95% CI, 1.37-2.95; *P* < .001) were associated with increased odds of diabetes. Regional TB endemicity and diabetes spatial distributions showed that there is a lack of consistent geographical overlap between these 2 diseases (eg, TB cluster 4: 60 213 TB cases; 186.79 diabetes cases in 20 183.88 individuals; 0.93% diabetes prevalence; TB cluster 8: 47 381 TB cases; 180.53 diabetes cases in 22 449.18 individuals; 0.80% diabetes prevalence; TB cluster 9: 37 620 TB cases, 601.45 diabetes cases in 12 879.36 individuals; 4.67% diabetes prevalence).

**Conclusions and Relevance:**

In this study, identifying spatial clusters of diabetes on the basis of a nationally representative survey suggests that India may face different levels of disease severity, and each region might need to implement control strategies that are more appropriate for its unique epidemiologic context.

## Key Points

**Question:**

What factors characterize the geographical distribution, associated socioeconomic and behavioral covariates, and overlap with tuberculosis endemicity of diabetes in India?

**Findings:**

In this cross-sectional study of 803 164 individuals living in India, the observed spatial variation of diabetes revealed spatial clustering of diabetes prevalence, which was associated with behavioral and socioeconomic factors, such as income, consuming alcohol, and smoking tobacco. Despite evidence for diabetes-tuberculosis interaction at the individual level, there was a lack of consistent geographic overlap between both diseases at the national scale.

**Meaning:**

This study adds to known risk factors associated with diabetes and identifies areas where diabetes prevalence is concentrated, including where potential overlap with tuberculosis occurs.

## Teaser

This cross-sectional study identifies locations in India where diabetes prevalence is concentrated, examines the association of diabetes with sociodemographic and behavioral covariates, and uncovers where high regional tuberculosis endemicity overlaps with diabetes.

## Introduction

Diabetes has been identified by the World Health Organization as an important public health issue.^1^ The estimated global burden of diabetes in 2014 was 422 million adults, and it is expected to increase to 552 million by 2030.^1,2^ Interest regarding the biological and epidemiologic interactions between diabetes and other diseases has recently grown. For example, tuberculosis (TB) has been considered part of the spectrum of diabetes-associated diseases.^3^ Possible causes of the diabetes-TB interaction include impaired glucose tolerance associated with TB treatment, which potentially increases the risk of diabetes.^4^ However, the mechanisms underlining glucose intolerance and diabetes in individuals with TB infections are not completely understood and are still subject of study.^4,5^

India, the second-most populous country in the world with 1.3 billion residents,^6^ has the largest number of diabetes cases, with a prevalence of 7.8%.^7^ Tuberculosis also severely affects the Indian population, with an incidence of 2.79 million in 2016.^8^ Such high burdens of both TB and diabetes might increase the likelihood of disease interactions that could worsen mortality from both diseases.^5,9^

Lack of information regarding the interaction between diabetes and TB exists, particularly in India. First, previous studies have overlooked the roles of behavioral risk factors in the prevalence of diabetes at a large scale. Second, little attention has been paid to the spatial structures of diabetes.^10,11^ Third, few studies have examined questions regarding the association between regional TB endemicity and diabetes, mainly because of the lack of affordable, large-scale, dual testing for TB and diabetes.^5,12^ Moreover, spatial variations in the association between diabetes and TB and the behavioral and environmental risk factors for diabetes-TB cooccurrence have not been assessed.^4,13,14^ Traditional epidemiologic approaches have a limited capacity to fill these gaps, and new approaches, such as spatial analysis, might be suitable for understanding the structure of epidemiologic risk factors for diabetes and diabetes-TB coexistence.^15^

Against this background, we used a national survey of more than 800 000 individuals (the India Demographic Health Survey [DHS] 2015-16^16^) and the Revised National TB control program (RNTCP) data for 2014 in India^17^ to identify the locations where the burden of diabetes is clustered and the areas where the burden of diabetes-TB exists. We also aimed to assess the association of diabetes with regional TB endemicity after controlling for important covariates and to examine sociodemographic and behavioral factors associated with diabetes in India. We hypothesized that individuals living in areas with high TB exposure would have an increased likelihood of developing diabetes.

## Methods

### Data Sources

Data used in this study came from 2 sources. For diabetes status and covariates, we used the India DHS, 2015 to 2016, a cross-sectional survey conducted from January 2015 to December 2016.^16^ The DHS sample defined primary sample units (PSUs) proportionally to the population using a 2-stage cluster sample design. Our final analytic sample included women aged 15 to 49 years and men aged 15 to 54 years, excluding individuals with a self-reported diabetes status different from yes or no. This sample resulted in 803 164 individuals located in 28 388 PSUs. For TB prevalence, data came from the RNTCP for 2014, which is the official source of the Indian government for TB profiles.^17^ This study followed the Strengthening the Reporting of Observational Studies in Epidemiology (STROBE) reporting guideline for cross-sectional surveys.^18^

Data were obtained from existing public access sources. Therefore, no specific ethical considerations and approvals applied to this study. The institutional review board of the ICF reviewed and approved the India DHS, 2015 to 2016. Participation in the survey was voluntary, and DHS obtained written consent before each interview was conducted.^16^

### Outcome Variable

Self-reported diabetes status was used as the outcome variable for our study. We used the DHS question “has a doctor or other health professional ever told you that you had diabetes?” as the measure for diabetes, with possible answers no, yes, and do not know. Only individuals answering no or yes were included, which accounted for 98.09% of the unweighted total sample (796 306 of 811 808 individuals).^19^

### Covariates

Sociodemographic and behavioral covariates were obtained from the DHS data set and selected according to relevant references.^10,20^ We estimated the association of diabetes with TB endemicity through directed acyclic graphs^21^ (eFigure 1 in the Supplement) and model selection based on the variance inflation factor analysis (eTable 1 in the Supplement). A total of 7 covariates were included in our final model, as follows: sex, age, religion, marital status, alcohol consumption, smoking tobacco, and body mass index (BMI; calculated as weight in kilograms divided by height in meters squared); 4 control variables were added to control for confounding, including educational level, rural or urban residence, wealth index, and land travel friction. Body mass index classification was according to the World Health Organization standards, defining normal weight as a BMI of 18.5 to less than 25.0.^22^

Tuberculosis exposure levels were computed based on the 2014 district-wise report from the RNTCP,^17^ adjusting by population from the most recent census (2011) from the Office of the Registrar and Census Commissioner.^23^ District-level population and TB cases were combined into TB cases per 100 000 inhabitants. Results were interpolated into a continuous surface of TB exposure using Gaussian kernel from the centroids of the district polygons.^24^ The RNTCP^17^ uses a 3-tier system of national reference laboratories to provide standardized TB diagnostic services. Data contain annualized total detected TB events using smear-positive, smear-negative, and extrapulmonary cases. Smear-positive is defined as an individual with TB infection who can transmit the infection, and individuals with TB infections in locations other than their lungs are classified as extrapulmonary cases. Data are available in the RNTCP repository.^17^ Reclassification of TB exposure areas was conducted using the following quantiles: level 1, less than 210 cases per 100 000 inhabitants; level 2, 210 to 311 cases per 100 000 inhabitants; level 3, 312 to 411 per 100 000 inhabitants; and level 4, more than 411 cases per 100 000 inhabitants.

Average land travel friction per meter was included for each PSU using measurements from the Malaria Atlas Project.^25,26^ Average land travel friction per meter is a continuous surface that measures interconnectivity between places, with the value of each pixel representing a nominal overall speed of travel expressed in minutes required to travel 1 meter (lower values correspond to less time to travel and therefore to more accessible areas). This metric is based on 10 global-scale surfaces derived from remote sensing measurements and the optimal speed of travel, computed from roads, railways, topographical conditions, and types of land cover.^25,27,28^ The friction map used the most updated data (ie, until 2015) from the Open Street Map and Google roads projects. Primary sample units were overlaid with both surfaces to extract TB exposure and land travel speed at each PSU, then reclassified into quantiles and assigned to each individual.

### Spatial Analyses

A scan statistical analysis was implemented in the SaTScan software (SaTScan) to identify the locations where diabetes cases were clustered.^29^ The spatial scan statistic is a cluster-detection test able to identify geographic clusters. For each cluster, the likelihood ratio was computed assuming independent Bernoulli distribution of cases. Additionally, maps with the locations of diabetes clusters were generated, including a smoothed surface of self-reported diabetes prevalence by PSU. Also, we aggregated cases over areas with and without diabetes clusters.

The spatial association of diabetes with TB infection at the ecological level was mapped using a clustering analysis similar to that described earlier for the RNTCP 2014 district-wise TB report. Poisson-based scan statistics were used with TB cases and the census population. Finally, maps of TB clusters were generated along with the corresponding endemicity level surface using the MapboxGL.js (Mapbox) mapping framework.

### Statistical Analysis

To assess the diabetes-TB coexistence, we implemented a methodology used to investigate the interaction between malaria and HIV,^30^ with a logistic regression model adjusting for PSU-level effects. First, we conducted a bivariate analysis between diabetes status, the 7 covariates from the DHS, TB data, and travel friction data. Only covariates with significant confidence intervals and *P* < .05 were included in the final multivariable model. All statistical analyses were conducted using R version 3.5.2 (R Project for Statistical Computing) and the *survey* and *svydiags* packages.^31^ Statistical significance was set at *P* < .05, and all tests were 2-tailed. We weighted covariates and adjusted for the 2-stage cluster sample design according to recommendations from DHS.^32^ Further details of our specific analysis are included in eAppendix 1 in the Supplement.

## Results

### General Description

Among 803 164 individuals in the sample, 691 982 (86.2%) were women and 111 182 (13.8%) were men. The cohort had a mean (SD) age of 30.09 (9.97) years. Results of the India DHS (2015-2016) indicated a self-reported diabetes prevalence of 1.76% (14 109 of 803 164 individuals). Using the India RNTCP 2014, 1 405 864 TB cases were reported in 2014, with a TB exposure of 127 cases per 100 000 inhabitants. Table 1 describes aggregated counts and prevalence of responders who reported diabetes for each covariate level. Aggregation of diabetes prevalence by TB exposure levels showed similar self-reported diabetes prevalence, increasing slightly from 1.73% (4651 of 269 050) in areas less exposed to TB (ie, level 1) to 1.88% (2822 of 150 487) in the most exposed areas (ie, level 4). Analysis of sociodemographic distribution of diabetes indicated that the highest prevalence was found in the oldest population, in groups aged 45 to 49 years (4646 of 84 697 [5.49%]) and 50 to 54 years (624 of 8626 [7.24%]). Likewise, results indicated a higher prevalence in urban settings than in rural settings (7402 of 283 259 [2.61%] vs 6707 of 519 906 [1.29%]).

**Table 1.** Characteristics of the Self-reported Diabetes Population From Demographic Health Survey, 2015 to 2016

Risk factor	Individuals with diabetes, No. (%) (n = 14 109)	Individuals with no diabetes, No. (n = 789 055)
TB exposure level, cases per 100 000 inhabitants		
<210	4651 (1.73)	264 409
210-311	3597 (1.72)	205 118
312-411	3039 (1.74)	171 863
>411	2822 (1.88)	147 665
Age group, y		
15-19	483 (0.35)	138 601
20-24	605 (0.44)	137 868
25-29	1020 (0.78)	128 959
30-34	1429 (1.30)	108 765
35-39	2149 (2.08)	101 174
40-44	3153 (3.55)	85 634
45-49	4646 (5.49)	80 051
50-54	624 (7.24)	8002
Sex		
Women	11 716 (1.69)	680 266
Men	2393 (2.15)	108 789
Religion		
Hindu	10 857 (1.67)	637 430
Muslim	2252 (2.04)	107 883
Sikh	233 (1.76)	13 005
Other	767 (2.43)	30 736
Educational level		
>Secondary	1889 (1.75)	106 213
Secondary	6734 (1.72)	384 257
Primary	2156 (2.16)	97 706
No education	3331 (1.63)	200 877
Marital status		
Never	914 (0.46)	196 126
Currently	12 289 (2.13)	563 466
Formerly	906 (2.98)	29 463
BMI		
>30.0	2081 (5.86)	33418
25.0-29.9	3691 (3.40)	104747
18.5-24.9	4705 (1.20)	388529
<18.5	978 (0.71)	137116
Missing	2654 (13.89)	16456
Tobacco smoking		
Yes	536 (3.39)	15 266
No	13 574 (1.72)	773 789
Alcohol consumption		
Yes	1204 (2.92)	39 964
No	12 906 (1.69)	749 091
Place of residence		
Rural	6707 (1.29)	513 199
Urban	7402 (2.61)	275 857
Wealth index		
Richest	5135 (2.98)	167 021
Richer	3983 (2.32)	167 437
Middle	2285 (1.38)	163 188
Poorer	1542 (0.99)	154 082
Poorest	1164 (0.84)	137 327
Land travel friction, min/m		
<0.001	4480 (2.47)	177 010
0.001-0.003	4028 (2.09)	188 984
0.003-0.016	3813 (1.44)	260 666
>0.016	1789 (1.09)	162 395

Abbreviations: BMI, body mass index (calculated as weight in kilograms divided by height in meters squared); TB, tuberculosis.

### Spatial Clustering Analysis of Diabetes

The locations and characteristics of the 4 spatial clusters for diabetes cases are summarized in Figure, A and Table 2. These areas comprised 40.44% of the total diabetes cases (8706 of 14 109), and prevalence was 3.68% (5706 of 155 023) in cluster areas compared with 1.30% (8403 of 648 141) in noncluster areas. Prevalence of diabetes varied between clusters (cluster 3, east Orissa: 2.81% [330 of 11 758]; cluster 1, Andhra Pradesh and Telangana: 3.01% [1864 of 61 948]; cluster 2, Tamil Nadup and Kerala: 4.32% [3429 of 79 435]; cluster 4, Goa: 4.43% [83 of 1883]).

**Figure.**
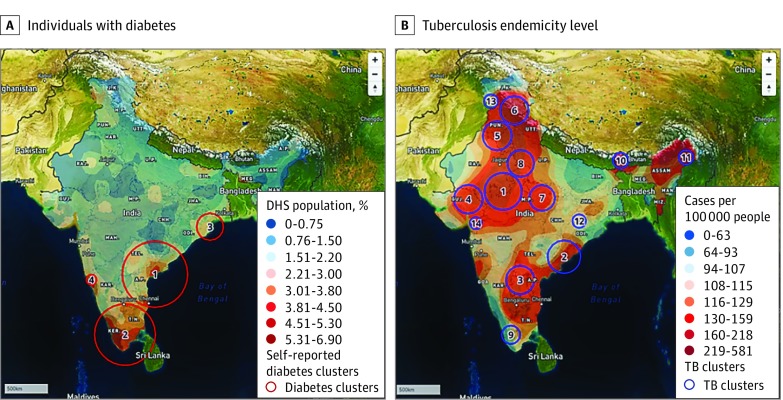
Clusters and Interpolated Surface Prevalence Levels of Diabetes and Tuberculosis Figures used map data from Mapbox, Open Street Map, DigitalGlobe, and their data sources. Data extracted from Open Street Map after September 2012 is licensed in terms of the Open Database License 1.0; it was previously licensed CC-BY-SA 2.0.

**Table 2.** Identified Self-reported Diabetes Clusters for the Demographic Health Survey, 2015 to 2016

Cluster	Radius, km	*P* value	Self-reported diabetes cases	Total population	Self-reported diabetes prevalence	Relative risk	TB exposure, mean (SD), cases per 100 000 inhabitants
1	373.55	<.001	1864	61 948	3.01	1.72	50.35 (14.57)
2	356.16	<.001	3429	79 435	4.32	2.47	160.06 (22.55)
3	147.96	<.001	330	11 758	2.81	1.61	113.06 (11.84)
4	58.09	<.001	83	1883	4.43	2.53	247.79 (3.63)
Total diabetes							
Hotspots	NA	NA	5706	155 023	3.68	NA	NA
No hotspots	NA	NA	8403	648 141	1.30	NA	NA
Overall	NA	NA	14 109	803 164	1.76	NA	NA

Abbreviations: NA, not applicable; TB, tuberculosis.

The number of diabetes cases and prevalence in each category, grouped by covariate and categorized between cluster and noncluster areas, was determined by additional aggregation analyses (eTable 2 in the Supplement). Diabetes prevalence by TB endemicity within the clusters ranged from 2.93% (1973 of 65 433) in level 1 areas to 5.13% (1179 of 21 786) in level 4 areas compared with the noncluster locations, which ranged from 1.27% (2159 of 167 764) in level 2 areas to 1.33% (2678 of 198 977) in level 1 areas.

### Spatial Clustering Analysis of TB Cases

We identified 14 TB clusters; their locations and the corresponding TB exposure surface are illustrated in Figure, B. These clusters contained 391 710 of 1 405 864 TB cases (27.86%) reported in the RNTCP 2014, mainly distributed across northern states (Jammu, Himachal Pradesh, Punjab, Uttar Pradesh, Delhi, Haryana, Gujarat, and Madhya Pradesh), northeastern states (Sikkim and Assam), and southern states in a lower proportion (Orissa, Andhra Pradesh, and Tamil Nadu). Reported TB cases and diabetes prevalence by each TB cluster appear in eTable 3 in the Supplement. Results of this analysis identified TB clusters with high TB and low diabetes in the northern half of India (cluster 4: 60 213 TB cases; 186.79 diabetes cases in 20 183.88 individuals; 0.93% diabetes prevalence; cluster 8: 47 381 TB cases; 180.53 diabetes cases in 22 449.18 individuals; 0.80% diabetes prevalence). Likewise, clusters with high TB and high diabetes were identified on the southwestern coast (cluster 9: 37 620 TB cases; 601.45 diabetes cases in 12 879.36 individuals; 4.67% diabetes prevalence).

### Association of Covariates With Diabetes

Tuberculosis exposure levels from the surrounding areas did not show a statistically significant association with diabetes at the individual level, except for slightly higher odds in TB level 3 areas in the adjusted model (odds ratio [OR], 1.16; 95% CI, 1.04-1.29; *P* = .03) (Table 3). In contrast, age, BMI, smoking tobacco, and alcohol consumption showed a positive association with reporting diabetes. For age, the youngest group (ie, aged 15-19 years) was used as the reference group, and all other groups showed a higher likelihood of diabetes, with the oldest group showing the highest odds (OR, 18.57; 95% CI, 13.67-25.23; *P* < .001).

**Table 3.** Unadjusted and Adjusted Models for Self-reported Diabetes According to Selected Biological and Sociodemographic Risk Factors

Risk factor	OR (95% CI)		*P* value
	Unadjusted	Adjusted	
TB exposure, cases per 100 000 inhabitants			
<210	1 [Reference]	1 [Reference]	NA
210-311	1.00 (0.91-1.09)	1.09 (0.99-1.20)	.53
312-411	1.01 (0.91-1.11)	1.16 (1.04-1.29)	.03
>411	1.09 (0.93-1.26)	1.02 (0.87-1.20)	.47
Sex			
Men	1 [Reference]	1 [Reference]	NA
Women	0.78 (0.71-0.86)	2.13 (0.86-5.19)	.10
Age group, y			
15-19	1 [Reference]	1 [Reference]	NA
20-24	1.26 (1.02-1.55)	1.08 (0.86-1.36)	.20
25-29	2.27 (1.85-2.80)	1.82 (1.43-2.32)	<.001
30-34	3.77 (3.03-4.69)	3.02 (2.32-3.92)	<.001
35-39	6.09 (4.96-7.48)	4.94 (3.82-6.39)	<.001
40-44	10.56 (8.49-13.14)	8.66 (6.60-11.36)	<.001
45-49	16.65 (13.32-20.82)	14.03 (10.67-18.50)	<.001
50-54	22.38 (17.28-29.00)	18.57 (13.67-25.23)	<.001
Religion			
Hindu	1 [Reference]	1 [Reference]	NA
Muslim	1.23 (1.13-1.33)	1.33 (1.20-1.48)	<.001
Sikh	1.05 (0.90-1.23)	0.74 (0.62-0.90)	<.001
Other or any	1.45 (1.27-1.69)	1.14 (0.96-1.35)	.14
Educational level			
>Secondary	1 [Reference]	1 [Reference]	NA
Secondary	0.99 (0.89-1.09)	1.15 (1.01-1.30)	<.001
Primary	1.24 (1.10-1.40)	1.19 (1.01-1.38)	.002
No education	0.93 (0.83-1.05)	0.83 (0.70-0.97)	<.001
Marital status			
Currently	1 [Reference]	1 [Reference]	NA
Formerly	1.41 (1.26-1.58)	0.99 (0.88-1.12)	.057
Never	0.21 (0.19-0.25)	0.74 (0.61-0.90)	.003
BMI			
18.5-24.9	1 [Reference]	1 [Reference]	NA
>30.0	5.15 (4.62-5.74)	2.44 (2.18-2.73)	<.001
25.0-29.9	2.91 (2.65-3.19)	1.66 (1.52-1.82)	<.001
<18.5	0.59 (0.52-0.66)	0.70 (0.62-0.78)	<.001
Tobacco smoking			
No	1 [Reference]	1 [Reference]	NA
Yes	2.00 (1.70-2.36)	3.04 (1.66-5.56)	<.001
Alcohol consumption			
No	1 [Reference]	1 [Reference]	NA
Yes	1.75 (1.55-1.98)	2.01 (1.37-2.95)	<.001
Place of residence			
Rural	1 [Reference]	1 [Reference]	NA
Urban	2.05 (1.90-2.22)	1.17 (1.06-1.29)	.001
Wealth index			
Poorest	1 [Reference]	1 [Reference]	NA
Poorer	1.18 (1.04-1.34)	1.02 (0.88-1.17)	<.001
Middle	1.65 (1.45-1.89)	1.14 (0.98-1.33)	.02
Richer	2.81 (2.45-3.21)	1.59 (1.36-1.86)	<.001
Richest	3.63 (3.17-4.15)	1.65 (1.40-1.95)	<.001
Land travel friction, min/m			
<0.001	1 [Reference]	1 [Reference]	NA
0.001-0.003	0.84 (0.75-0.95)	0.96 (0.85-1.09)	.02
0.003-0.016	0.58 (0.52-0.65)	0.82 (0.73-0.93)	.05
>0.016	0.44 (0.38-0.50)	0.75 (0.64-0.88)	.001

Abbreviations: BMI, body mass index (calculated as weight in kilograms divided by height in meters squared); NA, not applicable; OR, odds ratio; TB, tuberculosis.

Obesity and overweight showed higher odds (OR, 2.44; 95% CI, 2.18-2.73; *P* < .001; OR, 1.66; 95% CI, 1.52-1.82; *P* < .001, respectively), while individuals with underweight had lower odds of reporting diabetes (OR, 0.70; 95% CI, 0.62-0.78; *P* < .001) compared with the population with normal BMI (ie, 18.5 to <25.0). Smoking tobacco (OR, 3.04; 95% CI, 1.66-5.56; *P* < .001) and alcohol consumption (OR, 2.01; 95% CI, 1.37-2.95; *P* < .001) also resulted in increased odds of reporting diabetes. Sex did not have a statistically significant association after controlling for other cofactors (women: OR, 2.13; 95% CI, 0.86-5.19; *P* = .10).

Individuals living in urban settings showed increased odds of reporting diabetes compared with individuals living in rural areas (OR, 1.17; 95% CI, 1.06-1.29; *P* = .001). The wealth index was referenced to the poorest category and resulted in increased odds for those in the richest category (OR, 1.65; 95% CI, 1.40-1.95; *P* < .001) and the richer category (OR, 1.59; 95% CI, 1.36-1.86; *P* < .001). Lastly, living in areas with higher land travel friction (ie, >0.016 min/m) showed lower odds of diabetes (OR, 0.75; 95% CI, 0.64-0.88; *P* = .001). An extended description of these results can be found in eAppendix 2 in the Supplement.

## Discussion

Integrating a national survey data set that included more than 800 000 participants with a spatial clustering detection method, we found that diabetes was clustered in specific locations, mostly along the southern coastline of India. Moreover, our results suggested that there was a lack of consistent geographic overlap between diabetes and TB at the national scale. Whereas diabetes cases were concentrated in the southern part of the country, the northern part of India showed the highest burden of TB.

The highly dense areas of self-reported diabetes in southern India found in this study are consistent with other studies, indicating a growing epidemic with the highest prevalence in urban communities in the south, attributed to India’s rapid globalization and disparities in the ethnographic susceptibility to diabetes between populations in the northern and the southern states.^33^ Differences in dietary habits and sedentary lifestyle affect mostly the population located in the southern part of the country, which has been previously reported to be more genetically susceptible to diabetes compared with those in the north, which has a higher proportion of nonindigenous residents.^34^ Conversely, TB clustering analysis indicated a high burden of TB in north India, a region where diabetes prevalence was lower compared with southern regions. This could be associated with lower winter temperatures in the northern regions and increased seasonal variation of TB incidence^35^ because of seasonal respiratory infections that facilitate transmission, cause a delay in diagnosis, and generate a lack of vitamin D, which is a factor that increases the risk of TB infection.^36^

Although there is evidence of diabetes-TB interaction at the individual level, our results suggest a lack of consistency of this association at the ecological level.^37^ The overlap between TB and diabetes at a national scale has been found in areas where diabetes prevalence is more than 7%.^38^ Our analysis of covariate association for diabetes clusters and nonclusters showed increased odds of diabetes in areas with higher levels of TB exposure only within diabetes hotspots. This result could suggest that the potential lack of association of TB with diabetes at the national level was associated with the areas with a low prevalence of diabetes and high TB. Nevertheless, our geographic distribution of diabetes clusters and TB endemicity identified some partial overlapping in southern states.

Furthermore, we found that regional TB endemicity exposure did not increase the likelihood of reporting diabetes after controlling for important confounders consistently. Conversely, association analysis indicated that age, religion, economic status, having overweight or obesity, and consuming alcohol were risk factors associated with diabetes in this study and in previous studies.^10,39^ The association of smoking with diabetes was not reported in rural India and Bangladesh, and our findings reported an association between smoking and self-reported diabetes. Reasons for these results could be the inclusion of hypertension, physical activity, and TB endemicity, which are potential effect modifiers.^20,40,41,42^ Finally, higher wealth index and education were associated with higher odds of diabetes, which is also associated with a lower risk of TB in some regions in India,^43^ indicating that socioeconomic inequality could be contributing to the lower coexistence of high prevalence for both diseases at the national level.

The odds of diabetes were higher among individuals living in urban areas compared with rural communities. This finding is similar to previous reports among the rural and urban population in India.^33^ However, the prevalence in rural areas could increase as a result of the socioeconomic dynamics happening in these areas.^33,40^ Likewise, land travel friction was associated with the likelihood of reporting diabetes. Our results suggest that individuals living in areas with the highest land travel friction (>0.016 min/m), have 20% to 30% lower odds of reporting diabetes than individuals living in more accessible places. This result could be associated with different lifestyles in less accessible areas (ie, rural settings), where there may be more exposure to physical activities.^20^

### Limitations

Despite the strengths of our study, several limitations are worth noting. First, our main outcome was estimated from self-reported diabetes status from the DHS questionnaire, which could lead to the exclusion of individuals with diabetes who lack a clinical diagnosis. Other studies have used glucose level biomarkers available in the DHS for selected populations.^40^ The drawbacks of using this approach include lack of proper protocol for diabetes by the DHS guidelines. Only individuals who had glucose levels greater than 200 mg/dL (to convert to millimoles per liter, multiply by 0.555) were referred for diabetes screening, and reported fasting before the blood sample also relied on self-reported data. The use of plasma glucose levels for diabetes diagnosis requires retesting to assure the plasma glucose is unequivocally elevated. Epidemiologic studies often overclassify the population with diabetes by 25% (with false-positives) because of the lack of proper protocol.^44^ We assumed that self-reported diabetes would be a fairly accurate outcome because it relies on clinical confirmation, and self-reported data for health outcomes has been validated by multiple studies in India from the DHS survey.^45,46,47^ Furthermore, the DHS, 2015 to 2016, increased the quality of self-reported data by including standardized instruments and in-person interviews conducted in the local language.^48^

In addition to underdiagnosis, the DHS sample included only individuals aged 15 to 54 years, excluding most older individuals with a higher prevalence of diabetes. A similar study conducted in India using biomarkers from the DHS, 2015 to 2016, obtained a higher prevalence than the prevalence we derived from self-reported data.^48^ The magnitude of the difference (2.90% vs 1.76%) is on the order of expected values reported by several assessments of self-reported health outcomes.^45^ This difference also accounts for the lack of awareness of diabetes, especially in rural areas, where self-reported data can be flawed owing to low educational and socioeconomic status. Nonetheless, the association between the odds of high glucose and socioeconomic covariates remains consistent with what we found in our study.^48,49^ Finally, self-reported diabetes has been proposed as a reasonably good approximation of diabetes, particularly in population-based studies (eAppendix 3 in the Supplement).^49,50,51^

Although our work makes use of the most complete and contemporary databases for diabetes and TB in India, this study relied on TB exposure estimated in locations where the DHS was conducted. Tuberculosis cases were interpolated from district-wise cases confirmed by the RNTCP, making our endemicity level-map reliable but aggregated at different levels (eFigure 2 in the Supplement). Consequently, we assumed that TB exposure was highly correlated with TB infection, which could be inaccurate in large aggregated areas. Additionally, our regression model accounted only for the stratified 2-stage cluster sample design provided by the DHS (2015-2016). More advanced analysis to include multilevel modeling for complex survey data could adjust better for the cross-level effects at PSU and district levels. Therefore, the results of the association analysis of the coexistence of diabetes-TB should be interpreted with caution.

Despite these limitations, this study was the first that we know of to implement spatial analysis to investigate geographic structures of diabetes and to identify locations where a high prevalence of diabetes and TB coexist. India faces different intensities of diabetes and TB among districts, and each region might need to implement control strategies more appropriate for their unique contexts. This will result in more effective policies for reducing the burden of these diseases, especially in populations with social development and epidemiologic needs.

## Conclusions

In this study, the observed spatial variation of diabetes highlighted the existence of spatial clusters of diabetes at different scales with heterogeneities at regional scales in India, associated with behavioral and environmental covariates and partially overlapping with TB endemicity in areas with a high prevalence of diabetes. Our results highlight the need for more investment in early detection of diabetes, identification of populations at risk, and education about healthy life habits in the areas with high burdens. Identifying diabetes in individuals with early TB symptoms should be encouraged, especially in southern states, where diabetes prevalence is high, making the collision between both diseases highly likely.

## Notes

Supplement. **eAppendix 1. **Supplementary Methods **eAppendix 2. **Supplementary Results **eAppendix 3. **Supplementary Discussion **eTable 1. **Variance Inflated Factor Model Selection Results **eTable 2. **Description of Risk Factor Covariates by Cluster and Noncluster of Self-reported Diabetes and Proportions of the India DHS 2015-16 Sample **eTable 3. **Identified Tuberculosis Clusters From the Revised National TB Control Program (RNTCP) 2014 **eFigure 1. **Directed Acyclic Graph for Self-reported Diabetes and Behavioral and Environmental Risk Factors **eFigure 2. **Geographic Distribution of Population Included in the Analysis **eReferences. **
