# Error in Affiliations

**DOI:** 10.1001/jamanetworkopen.2020.10311

**Published:** 2020-05-28

In the Original Investigation titled “Geographic Variation and Associated Covariates of Diabetes Prevalence in India,”^1^ published May 1, 2020, there was an error in the author affiliations. Dr Jia’s affiliations should have included the International Initiative on Spatial Lifecourse Epidemiology (ISLE), Hong Kong, China. This article has been corrected.^1^
