# Supplementary material for: Geographic Variation and Associated Covariates of Diabetes Prevalence in India
Source: JAMA Netw Open. 2020 May 1;3(5):e203865. doi: 10.1001/jamanetworkopen.2020.3865 (PMC7195623; doi:10.1001/jamanetworkopen.2020.3865)
Supplement: Supplement. — eAppendix 1. Supplementary Methods eAppendix 2. Supplementary Results eAppendix 3. Supplementary Discussion eTable 1. Variance Inflated Factor Model Selection Results eTable 2. Description of Risk Factor Covariates by Cluster and Noncluster of Self-reported Diabetes and Proportions of the India DHS 2015-16 Sample eTable 3. Identified Tuberculosis Clusters From the Revised National TB Control Program (RNTCP) 2014 eFigure 1. Directed Acyclic Graph for Self-reported Diabetes and Behavioral and Environmental Risk Factors eFigure 2. Geographic Distribution of Population Included in the Analysis eReferences. [file jamanetwopen-3-e203865-s001.pdf]

Supplementary Online Content

Hernandez AM, Jia P, Kim H-Y, Cuadros DF. Geographic variation and associated covariates of diabetes prevalence in India. *JAMA Netw Open*. 2020;3(5):e203865. doi:10.1001/jamanetworkopen.2020.3865

eAppendix 1. Supplementary Methods
eAppendix 2. Supplementary Results
eAppendix 3. Supplementary Discussion
eTable 1. Variance Inflated Factor Model Selection Results
eTable 2. Description of Risk Factor Covariates by Cluster and Noncluster of Self-reported Diabetes and Proportions of the India DHS 2015-16 Sample
eTable 3. Identified Tuberculosis Clusters From the Revised National TB Control Program (RNTCP) 2014
eFigure 1. Directed Acyclic Graph for Self-reported Diabetes and Behavioral and Environmental Risk Factors
eFigure 2. Geographic Distribution of Population Included in the Analysis
eReferences.

This supplementary material has been provided by the authors to give readers additional information about their work.

© 2020 Hernandez AM et al. *JAMA Network Open.*

eAppendix 1: Supplementary Methods

Demographic Health Survey (DHS) Sample Design

The DHS India 2015-16 has as its primary aim to provide valuable data to prevent and measure emerging issues on health and family welfare for public health decision making. It has a clinical, anthropometric, and biochemical (CAB) component designed to estimate prevalence of malnutrition, anemia, hypertension, HIV, and high blood glucose levels using a series of biomarkers, interviews, and measurements. The DHS India 2015-16 target population is women and men of reproductive age and children living on households. In our study, representative sample refers to population of women and men on reproductive or working ages. Our study estimates self-reported diabetes prevalence, and associated risk factors on women, and men of these age groups. Reproductive age women are considered aged from 15 to 49, and men from 15 to 54 years.

The Demographic Health Survey (DHS) defines primary sample units (PSUs) proportionally to the population using a two-stage cluster sample design. Then, approximately 20-30 households, which are previously identified to fulfill inclusion criteria, are randomly selected in each PSU at the central office of the Ministry of Health and Family Welfare to prevent potential bias. For the India DHS 2015-16 (NFHS-4), 14 field agencies collected information from 28,388 PSUs (19,917 rural and 8,471 urban units) during 20th January 2015 and 4th December 2016, which resulted in a cross-sectional sample of 601,500 households with 699,686 women and 112,122 men[1]. The survey was conducted in local language by implementing a computer assisted personal interview on household sociodemographic characteristics and health status of all household members and, for the purpose of covering as many people as possible, the visitors who stayed in the households the night before and were still present during the interview [1]. All information including PSU locations is available on the DHS webpage in individual data files for households, women, and men [2,3].

DHS India 2015-16 used the stratified two stage sampling technique in order to make sure that the sample is representative for the national target population. This technique is used to allow every individual on the target population to have a chance of being selected and included in the survey, and to prevent selection bias. DHS India 2015-16 used a sampling frame, which corresponded to a list of all PSUs (also called Enumeration Areas (EAs) or Sampling clusters), and the population of each PSU within the country. The India Census 2011 served as the sampling frame for the selection of the PSUs to identify the 100% of the target population.

After the identification of PSUs and population in the country, a stratification process took place to classify PSUs into homogeneous subgroups. This process allows to identify a representative sample for each subgroup. DHS India 2015-16 used grouping classification of PSUs located in rural and urban settings and included in the dataset of the survey. Based on the sampling frame stratification, the sample size was designed by the DHS India 2015-16 experts to define the PSUs selected for the survey, and the sampling for the number of households or individuals needed on these PSUs, to have statistically reliable results, and a representative of the country as a whole.

PSUs are areas which divide the sampling framework. For the DHS 2015-16, PSUs have different definitions for rural and urban settings. In rural areas, PSUs correspond to villages or part of villages, and in urban settings PSUs correspond to Census Enumeration Blocks (CEB). DHS India 2015-16 aggregated PSUs with less than 40 households to the nearest larger PSU. Rural and urban settings had similar criteria for selection of PSUs. As explained before, within each rural stratum, villages were selected from the sampling frame with probability proportional to size (PPS). In urban areas, CEB information was obtained from the Office of the Registrar General and Census Commissioner. CEBs were sorted according to the percentage of the population in each CEB, and sample CEBs were selected with PPS sampling.

In every selected rural and urban PSU, a complete household mapping and listing operation was conducted prior to the main survey. Selected PSUs with an estimated number of at least 300 households were segmented into segments of approximately 100-150 households. Two of the segments were randomly selected for the survey using systematic sampling with probability proportional to segment size. Therefore,

© 2020 Hernandez AM et al. *JAMA Network Open.*

India DHS 2015-16 clusters are either a PSU or a segment of a PSU. In the second stage, in every selected rural and urban cluster, 22 households were randomly selected with systematic sampling.

Finally, in the stratified two-stage sampling, PSUs were selected with random probability proportionally on the size (PPS), which means that PSUs with larger population are more likely to be selected for the survey. As a result, the PSUs locations (eFigure1 B) are representative of the target population density of India estimated from the Census 2011. PPS results in areas with fewer population having less households included in the survey. However, some mortality and fertility rates estimations require a minimum number of households or individuals to be included to estimate reliable results, regardless of the population size. Considering this constraint, oversampling of small regions, and under sampling of large regions is performed on the DHS India 2015-16. In order to restore representativeness of the sample and to prevent bias, DHS India 2015-16 includes sampling weights at household and individual levels. All weights and survey design were included in our statistical analysis as recommended by the DHS data analysis guidelines from the DHS program.

Revised National Tuberculosis Control Program (RNTCP) Reporting Process

This program utilizes a three-tier system of national reference laboratories, intermediate reference laboratories, and designated microscopy centers equipped for providing internationally standardized tuberculosis (TB) diagnostic services [4]. Data contain annualized total detected TB events using smear positive, smear negative and extra pulmonary cases. An smear positive is considered as an individual TB infected who can transmit the infection, and individuals with the TB infection in other than lungs are classified as extra pulmonary cases [5]. Data is available on the online repository of the Revised National Tuberculosis Control Program (RNTCP) [4].

Model selection and covariates description

Model selection for the risk estimation of self-reported diabetes status was conducted by using a directed acyclic graph (DAG), to represent a structure of conditional independence among the selected covariates. DAGs describe statistical relationships among risk factors using a representation of nodes and directed edges that consider potential influence among covariates [6]. Moreover, DAGs are useful for elucidating causal paths and to control for different source of systematic bias on statistical models, especially for confounding among an exposure and outcome variables. eFigure1 describe the relation model between self-reported diabetes and TB endemicity exposure, including individual and PSU level covariates of interest that have shown relation with diabetes among the literature [7,8].

DAGs has shown to be useful in determining the variables needed to control for effects of confounding when estimating the causal relation between exposure factors and the outcome. Statistical causal relations in DAGs are mathematically determined by adjusted and unadjusted paths. Paths are sequences of nodes connected by edges regardless of arrowhead direction [6]. eFigure1 describe control variables that are needed to control for systematic confounding between diabetes and TB endemicity level, considering the limitations of availability of data and other individual risk factors of interests. Adjusted paths were identified using the ggdag package in R statistical software and represented using the dashed arrow notation and squared nodes. Finally, for accounting unmeasured environmental adjusting factors we used the land travel friction covariate from the atlas malaria project, and we adjusted by the wealth index, place of residence, and educational level factors. Individual level covariates of interest are included in the bottom of the main relation and describe impacts among them with solid directed edges.

Finally, the individual covariates were classified using appropriate levels: gender (male or female), age (by quinquennial age group), religion (Hinduism, Islam, Sikh, or others), highest education level (no education, elementary, secondary, or higher), marital status (never married, currently married, or formerly married), alcohol use (yes or no), smoke tobacco (yes or no), weight status (underweight, normal,

© 2020 Hernandez AM et al. *JAMA Network Open.*

overweight, or obese), type of place of residence (urban or rural) and household wealth index (by quintiles). Taking into account that DHS individuals include population under 18, an slight modification for weight status was defined based on body mass index (BMI, unit: kg/m2) according to WHO standards, for under 18 population group: 'underweight', 'normal', 'overweight', and 'obesity' were defined as WHO-recommended sex-age-specific BMI <15th percentile (corresponding to BMI=18.5 at age 18 years), ≥15th but <85th (corresponding to BMI=25 at 18), ≥85th but <97th (corresponding to BMI=30 at 18), and ≥97th, respectively [9,10].

Wealth index covariate is computed at household level from a set of factors included in the India DHS 2015-16 survey. The wealth index composite measure of a household's cumulative living standard. Household's ownership of selected assets are considered on this metric such as televisions and bicycles; materials used for housing construction; and types of water access and sanitation facilities [11]. Finally, a Principal Component Analysis PCA algorithm is used to identify contribution of this main components of the living standards of the target population and included at household and individual within the available datasets of the survey.

Spatial Analyses Additional Notes

An advantage provided by the spatial scan statistics is the ability to account for the uneven distributions of the sampled population. Scan statistics scanned a circular window that spanned the study region. The radius of the circle was changed continuously, so that it could take any value from 0% up to the default value of 50% of the sampled population inside the window. SatScan maximum radii size was set to 50 km for the self-reported diabetes clustering analysis considering the high density of PSUs included in the study. Similarly, TB clustering analysis maximum radii was set to 250 km to avoid high rate of overlapping. The numbers of observed and expected self-reported diabetes cases within and outside the circular window were then compared with the likelihood L0 under the null hypothesis of spatial randomness. The circular windows with the highest likelihood ratio values were identified as clusters. An associated p-value of the statistics was then determined through Monte Carlo simulations and used to evaluate whether self-reported diabetes cases were randomly distributed in space or not.

Spatial data mapping was performed using Mapbox GL JS v 1.3.1 [12]. All statistical analyses were conducted using R statistical software [13] and the survey and svydiags packages [14]. We weighted individual and PSU-level data and adjusted for two-stage cluster sample design according to recommendations of DHS [15].

eAppendix 2: Supplementary Results

Model selection Analysis results

Complementarily to the DAG analysis, we performed a Variance Inflated Factor (VIF) stepwise model selection to account for multicollinearity among the selected variables using the svydiags package on R to account for the stratified two-stage sampling followed by the India DHS 2015-16 showed in the eTable1. VIF stepwise model selection used the correlation matrix computed over the samples, and measure collinearity using a multiple regression, and taking the ration of all given models divide by the variance of the single coefficients for each variable if it were fitted individually. All variables less than five (5.00) VIF score were allowed to be included in the adjusted model. For our specific DAG, any variable showed high correlation among the dataset and all adjusted paths and individual risk factors for diabetes were included in the final model.

Overall prevalence and demographical characteristics

Individual-level covariates association indicated that the highest prevalence of self-reported diabetes by age group was found in the oldest population between the ages of 45-49 for females (5.52%), and 50-54 for males (7.24%). Additionally, population reported as practicing 'other or any religion' was the group with highest prevalence for both genders, (females 2.24%, males 3.77%), followed by Muslims (females 2.04%, males 2.08%), Sikh (females 1.71%, males 2.08%), and Hinduists (females 1.61%, males 2.09 %). On the contrary, self-reported diabetes aggregated by educational level showed a disparity between genders, with highest prevalence on the population with 'primary' studies for females (2.14%), and 'Higher' studies for males (2.56%). Finally, BMI covariate was included mostly for females due to high proportion of missing values for males (98% of total male population missing BMI measurement). This covariate showed a distribution of self-reported diabetes prevalence of obese - 5.86%, overweight - 3.40%, normal - 1.20%, and finally underweight population - 0.71%.

PSU-level covariate place of residence showed higher prevalence in urban settings (females urban 2.59% vs. females rural 1.21%, and males urban 2.72% vs. males rural 1.80%). Moreover, wealth index factor showed also highest prevalence in the richest population for both females and males (2.91% and 3.29% respectively). Finally, average land travel friction per meter also showed highest prevalence in the areas with less friction (females 2.41% and males 2.83%, in areas with less than 0.001 min per meter) compared to areas with higher friction (females 1.02% and males 1.53%).

Association Analyses Expanded Results

Additional associated covariates in the analysis were religion and marital status. Religion showed an increasing risk for diabetes in the 'Muslim' category (OR=1.33; 95% CI=1.20–1.48), and decreasing risk for 'Sikh' (OR=0.74; 95% CI=0.62–0.88), and not association for the 'Other or Any Religion' category compared with the Hinduist population. Marital status was also associated with the risk of developing diabetes, with lower risk for the 'Never Married' individuals (OR=0.74; 95% CI=0.61–0.90) compared to the 'Currently Married' population.

© 2020 Hernandez AM et al. *JAMA Network Open.*

eAppendix 3. Supplementary Discussion

Additional notes on limitations of the study

Important drawback of using plasma glucose levels for diabetes status is that diabetes diagnosis requires careful substantiation with retesting to assure the plasma glucose is unequivocally elevated. Epidemiological diabetes studies often overclassify the population with diabetes, by 25% of false positive cases due to lack of repeat testing [55]. We assumed that the 'currently has diabetes' variable is a more accurate outcome because it relies on clinical confirmation which is often based on retesting and includes other clinical aspects associated with diabetes. Self-reported diabetes will likely underestimate diabetes prevalence as a large fraction of population with diabetes are unaware of their disease status [56]. In our study, the diabetes prevalence resulted in 1.76%, a lower estimation than the 7.8% estimated for India [7]. In addition to under-diagnosis, the DHS sample included only individuals aged 15–59 years and excluded older populations with higher prevalence of diabetes. However, despite the use of this indirect measure for the TB endemicity and potential effects of different area unit scaling effects [57], we performed complementary clustering analyses, which was not affected by aggregation and was robust to indicate low geographic overlaps between both diseases.

© 2020 Hernandez AM et al. *JAMA Network Open.*

eTable 1. Variance Inflated Factor Model Selection Results

Risk Factor	Level	VIF score
TB Exposure	Level 1 (<= 209)	Ref.
	Level 2 (210 - 311)	1.351
	Level 3 (312 - 411)	1.325
	Level 4 (>= 412)	1.323
Age Group	15-19	Ref.
	20-24	2.463
	25-29	2.960
	30-34	2.798
	35-39	2.728
	40-44	2.649
	45-49	2.620
	50-54	NA
Religion	Hindu	Ref.
	Muslim	1.050
	Sikh	1.056
	Other Religion	1.026
Educational Level	Higher	Ref.
	Secondary	3.241
	Primary	2.389
	No education	3.808
Marital Status	Never Married	2.298
	Currently Married	Ref.
	Formerly Married	1.047
Body Mass Index	Obese	1.106
	Overweight	1.181
	Normal	Ref.
	Underweight	1.167
Smoke Tobacco	Yes	1.354
	No	Ref.
Alcohol	Yes	1.807
	No	Ref.
Place of residence	Rural	Ref.
	Urban	1.712
Wealth Index	Richest	2.060
	Richer	1.649
	Middle	Ref.
	Poorer	1.599
	Poorest	1.690
Land Travel Friction quantiles	1st (< 0.001)	Ref.
	2nd (0.001-0.003)	1.642
	3rd (0.003-0.016)	2.034
	4th (> 0.016)	2.013

© 2020 Hernandez AM et al. *JAMA Network Open.*

eTable 2. Description of Risk Factor Covariates by Cluster and Noncluster of Self-reported Diabetes and Proportions of the India DHS 2015-16 Sample

Risk Factor	Level	Cluster Diabetes (No.)	(%)	Non diabetes Cluster (No.)	No-cluster Diabetes (No.)	(%)	Non diabetes No-Cluster (No.)
TB Exposure	Level 1 (<= 209)	1,973	(2.93)	65,433	2,678	(1.33)	198,977
	Level 2 (210 - 311)	1,438	(3.71)	37,353	2,159	(1.27)	167,764
	Level 3 (312 - 411)	1,116	(4.32)	24,745	1,923	(1.29)	147,117
	Level 4 (>= 412)	1,179	(5.13)	21,786	1,643	(1.29)	125,879
Age Group	15-19	163	(0.77)	21,020	320	(0.27)	117,581
	20-24	207	(0.86)	23,748	397	(0.35)	114,120
	25-29	398	(1.57)	24,982	623	(0.60)	103,976
	30-34	497	(2.26)	21,505	932	(1.06)	87,261
	35-39	882	(4.03)	20,990	1,267	(1.56)	80,183
	40-44	1,317	(7.06)	17,335	1,835	(2.62)	68,299
	45-49	1,953	(9.85)	17,866	2,694	(4.15)	62,185
	50-54	289	(13.40)	1,871	335	(5.18)	6,133
Religion	Hindu	4,576	(3.53)	124,925	6,281	(1.21)	512,506
	Muslim	675	(4.48)	14,388	1,577	(1.66)	93,495
	Sikh	2	(5.39)	35	231	(1.75)	12,971
	Other Religion	453	(4.35)	9,969	314	(1.49)	20,766
Educational Level	Higher	842	(2.83)	28,951	1,046	(1.34)	77,262
	Secondary	2,871	(3.56)	77,849	3,863	(1.25)	306,409
	Primary	915	(5.68)	15,193	2,253	(2.66)	82,513
	No education	1,078	(3.80)	27,324	1,241	(0.71)	173,554
Marital Status	Never Married	377	(1.08)	34,404	536	(0.33)	161,720
	Currently Married	4,955	(4.39)	107,798	7,334	(1.58)	455,667
	Formerly Married	374	(4.99)	7,115	533	(2.33)	22,351
Body Mass Index	Obese	842	(7.53)	10,338	1,239	(5.09)	23,114
	Overweight	1,591	(5.43)	27,713	2,099	(2.65)	77,198
	Normal	1,836	(2.67)	66,848	2,868	(0.88)	322,491
	Underweight	252	(1.32)	18,886	726	(0.61)	118,494
	Missing (NA)	1,184	(4.43)	25,532	1,471	(1.47)	98,441
Smoke Tobacco	Yes	295	(6.85)	145,305	241	(2.10)	11,255
	No	5,411	(3.59)	4,012	8,162	(1.28)	628,482
Alcohol	Yes	700	(5.57)	137,438	504	(1.76)	28,085
	No	5,006	(3.51)	11,879	7,899	(1.27)	611,653
Place of residence	Rural	2,343	(2.83)	80,517	4,365	(1.00)	432,682
	Urban	3,363	(4.66)	68,800	4,038	(1.91)	207,056
Wealth Index	Richest	2,184	(5.26)	39,300	2,951	(2.26)	127,721
	Richer	1,910	(3.99)	45,973	2,073	(1.68)	121,464
	Middle	1,039	(2.73)	8,039	1,246	(0.98)	126,202
	Poorer	395	(2.03)	19,019	1,147	(0.84)	135,063
	Poorest	178	(2.17)	36,986	986	(0.76)	129,288
Land Travel Friction	1st (< 0.001)	2,210	(4.82)	43,618	2,270	(1.67)	133,392
	2nd (0.001-0.003)	1,647	(3.66)	43,402	2,381	(1.61)	145,582
	3rd (0.003-0.016)	1,382	(3.04)	44,117	2,431	(1.11)	216,548
	4th (> 0.016)	467	(2.50)	18,180	1,321	(0.91)	144,216
**Total counts**		**5,706**	**(3.68)**	**149,317**	**8,403**	**(1.30)**	**639,738**
**DHS target population Denominator**		**155,023**			**648,141**		

© 2020 Hernandez AM et al. *JAMA Network Open.*

eTable 3. Identified Tuberculosis Clusters From the Revised National TB Control Program (RNTCP) 2014.

ID	RADIUS (km)	P_VALUE	Observed TB Cases	Relative Risk TB	Self-reported Diabetes (%)
1	199.14	<0.001	58,022	1.39	1.15
2	185.01	<0.001	32,561	1.2	2.31
3	161.85	<0.001	21,657	1.27	1.60
4	156.53	<0.001	60,213	1.5	0.93
5	149.58	<0.001	45,474	1.26	1.32
6	142.97	<0.001	20,622	1.77	1.64
7	140.03	<0.001	22,249	1.3	1.47
8	140.03	<0.001	47,381	1.24	0.80
9	106.96	<0.001	37,620	1.24	4.67
10	80.93	<0.001	10,809	1.35	2.18
11	74.69	<0.001	6,867	1.89	0.92
12	73.82	0.01	5,434	1.07	1.37
13	68.2	<0.001	4,673	1.36	2.76
14	64.11	<0.001	18,128	1.73	1.66
**Total TB Hotspots**			**391,710**	**1.35**	**1.63**
**Total TB No Hotspots**			**1,014,154**		**1.79**
**Total TB**			**1,405,864**		**1.76**

© 2020 Hernandez AM et al. *JAMA Network Open.*

eFigure 1. Directed Acyclic Graph for Self-reported Diabetes and Behavioral and Environmental Risk Factors

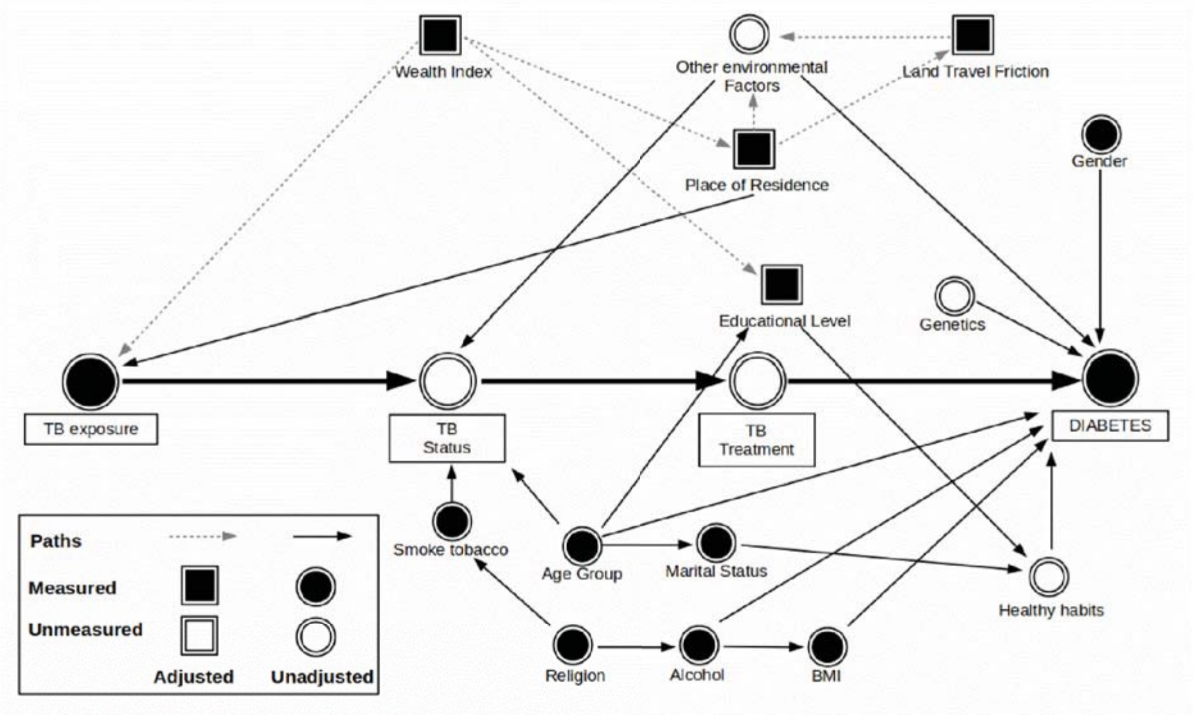

© 2020 Hernandez AM et al. *JAMA Network Open.*

eFigure 2. Geographical Distribution of Population Included in the Analysis

(A) Demographic and Health Survey sample data points (Primary Sample Units - PSU) conducted in India DHS 2015-16; (B) India Revised National Tuberculosis Control Program Districts 2014.

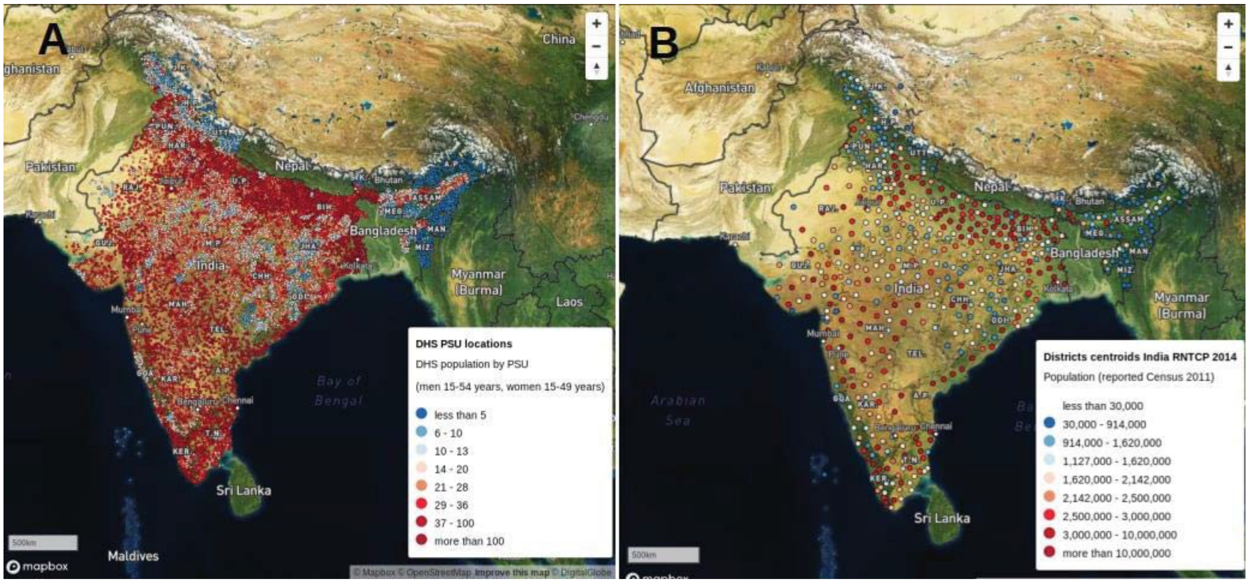

© 2020 Hernandez AM et al. *JAMA Network Open.*

eReferences

1. International Institute for Population Sciences - Deemed University (Mumbai), Government of India - Ministry of Health and Family Welfare National. National Family Health Survey - 4: 2015-16 , India Fact Sheet. In:2016:1--6.
2. The DHS Program. Demographic and Health Surveys (various) Datasets . In.
3. USAID. Standard Recode Manual for DHS 6. *DHS Publication.* 2013:1--7.
4. Central TB Division Directorate General of Health Services Ministry of Health and Family Welfare India. TB India 2014, Revised National Tuberculosis Control Programme, Annual Status Report. In: Ministry of Health and Family Welfare, Nirman Bhavan, New Delhi; 2014:204.
5. Narula P, Sihota P, Azad S, Lio P. Analyzing seasonality of tuberculosis across Indian states and union territories. *Journal of Epidemiology and Global Health.* 2015;5(4):337-346.
6. Hernán MA, Cole SR. Invited Commentary: Causal diagrams and measurement bias. *American journal of epidemiology.* 2009;170(8):959-964.
7. Barik A, Mazumdar S, Chowdhury A, Rai RK. Physiological and behavioral risk factors of type 2 diabetes mellitus in rural India. *BMJ Open Diabetes Research and Care.* 2016;4(1):1-8.
8. Geldsetzer P, Manne-Goehler J, Theilmann M, et al. Diabetes and hypertension in India a nationally representative study of 1.3 million adults. *JAMA Internal Medicine.* 2018;178(3):363-372.
9. Khadilkar VV, Khadilkar AV. Revised Indian Academy of Pediatrics 2015 growth charts for height, weight and body mass index for 5-18-year-old Indian children. *Indian Journal of Endocrinology and Metabolism.* 2015;19(4):470.
10. World Health Organization (WHO). Body mass index - BMI. In.
11. The DHS Program. Demographic Health Survey (DHS) Official Website. In.
12. Mapbox. Mapbox GL JS API. (Satellite tiles by DigitalGlobe). In:2019.
13. R Development Core Team. R: A Language and Environment for Statistical Computing. In:2008.
14. Lumley T. Analysis of Complex Survey Samples. *Journal of Statistical Software.* 2004;9(1):1-19.
15. The DHS Program. How to Weight DHS Data in SPSS & SAS. In:2015.

© 2020 Hernandez AM et al. *JAMA Network Open.*
